# Supplementary material for: Patterns of Intron Gain and Loss in Fungi
Source: PLoS Biol. 2004 Nov 30;2(12):e422. doi: 10.1371/journal.pbio.0020422 (PMC532390; doi:10.1371/journal.pbio.0020422)
Supplement: Table S1 — Also available at http://genes.mit.edu/NielsenEtAl/. (4.3 MB ZIP). [file pbio.0020422.st001.zip › NielsenEtAl/html/265.html]

AN1023.1.NCU06347.1.MG06180.1.FG09721.1


```
 CLUSTAL W (1.82) Multiple Sequence Alignments - Introns Inserted


Sequence 1: NCU06347.1	410 aa
Sequence 2: MG06180.1	396 aa
Sequence 3: FG09721.1	397 aa
Sequence 4: AN1023.1	389 aa
Alignment Length: 420 aa
Number Identitical Residues: 212 aa
Alignment Score (without introns) 10751


MG06180.1 	MAP-RIEAQEIETYWNIFSARTNGSKFLTGEQAAPVLKNSGLRDDQLERVWDLADVDNDG
NCU06347.1	MPP-KIEAQEIETYWNIFSARTNGGKFLTGEQAAPVLKNSGLRDDQLERVWDLADVDNDG
FG09721.1 	MAP-KIEEQEIETYWNIFSTRTGGGKFLTGEQAAPVLKNSGLRDDQLERVWDLADVDNDG
AN1023.1  	MSNKKIEQWEVERYWEIFSSLAGGQPHLNNSQAASVLRNSRLRDDQLEKVWDLADLDGDG
          	*. .:**  *:* **:***: :.*  .*...***.**:** *******:******:*.**

MG06180.1 	NLDFEEFCVAMRVIFDILNG0EHADVPSTLPDWLVPESKAHLVQANRALTGKQVQFERVD
NCU06347.1	NLDFEEFCVAMRVIFDLLNG0EYADVPTTLPDWLVPESKAHLVQANRALTGKAPKFEQVE
FG09721.1 	NLDFEEFCVAMRIIFDILNG0EYADVPTTLPDWLVPESKAHLVQATQAITGKQVQFEQVE
AN1023.1  	ELDFEEFCVAMRLVFDLVNG0ELPQVPKSLPDWLVPESKAHLVQATRALSGGQEQFERIE
          	:***********::**::** * .:**.:****************.:*::*   :**:::

MG06180.1 	DDPDSPGLKDGFDWYMSPADKSKYESIYQENRDMRGEVSF1GALEDLYESLDVPDTDIRS
NCU06347.1	DEDDTPGLKDGFDWYMSPADKSKYEQIYQENRDMRGEVSF1AALQDLYDSLSVPDTDIRS
FG09721.1 	DDSEDLGLKDGFEWYMKPADKSKYEQIYQENRNARGEVDF1NALSDLYESLDVPDTDIRS
AN1023.1  	DEDSTPGLKDGFDWYMSPEDKAKYEEIYSANKNQRGEIAF1GSLEPLYESLDVPDTDIRS
          	*: .  ******:***.* **:***.**. *:: ***: *  :*. **:**.********

MG06180.1 	AWNLINPSAGPTINKDACLAFLHILNYRHEGYRIPRTVPASLRASFERNKIDYQVDKQ--
NCU06347.1	AWNLVNPSASSTINKDACLAFLHILNYRHEGFRIPRTVPASLRASFERNKITYNVNDP--
FG09721.1 	AWNLINPSAQSTINKDACLAFLHILNYRHEGFRIPRAVPASLRSSFERNQIDYQVDNQRT
AN1023.1  	AWNLVNPSAAPEINKDATLAFLHILNYRHEGYRIPRTIPASLRASFENNKIDYQLDSAR-
          	****:**** . ***** *************:****::*****:***.*:* *:::.   

MG06180.1 	-AASRWATKADDETSTGRKAKFGDQYLTRL---GRGSFKTSGTDFSSAQTDSEWEEVRLK
NCU06347.1	-SQSRWAQKADDETSTGRKAKFGDQYLTRL---GRSSFKTAGTDFSSEQTDDQWEEVRLK
FG09721.1 	GANSRWATKADDSTSTGRKAKFGDQYLTRL---GRSGFKTAGTDFSTEKTED-WEEVRLK
AN1023.1  	-PAQKWGTNGDTETSTGRKAKFGDTYLSRLGVAGKTSYTPKGTDFSDTIQDEEWEKVRLR
          	 . .:*. :.* .*********** **:**. :*: .:.. *****    :..**:***:

MG06180.1 	KQLAELDAKMASVEADAERRKGGKR-DSKP--ALVKRELEQLLDYKRKQLREIEEGKTKG
NCU06347.1	KQLAELDEKMAKVEEAANRRRGGKR-DTKP--ALVKRELEQLLDYKRKELRDLEEGKGKA
FG09721.1 	RKLQDLEEKVKKVEDIAERRKGGKR-DSKP--ALVKKELDQLLEYKRRELRELEEGTGKS
AN1023.1  	RELAELDAKLQAANKAVEGRKAGNRNDGRPNWVLIKKEALQLLEYKERELRELREG----
          	::* :*: *:  .:  .: *:.*:*.* :*. .*:*:*  ***:**.::**::.**    

MG06180.1 	QGGGSLKGIQDD~LQTVREQTDGLASHLRSRQQHLEELRRQIEDEKAGR~----------
NCU06347.1	AAGGSLKSVADD~LATVKEQVEGLEAHLRSRQDVLEQLKREIEEEKVGR2YLFDFISVKR
FG09721.1 	AAGGSLKSIQED~LQTVREQVEGLETHLRTREQVLAEIRREIEDEKRG-~----------
AN1023.1  	-----------N1VRTVGEQVDGLKNHLIQRKGVLEDLRREIEDERASR~----------
          	           : : ** **.:**  **  *:  * :::*:**:*: .            

MG06180.1 	----
NCU06347.1	HRKS
FG09721.1 	----
AN1023.1  	----
          	
```
